# Social Group Membership With Residents Mediates Reinforcement Outcomes for a Translocated Bird

**DOI:** 10.1002/ece3.74136

**Published:** 2026-08-20

**Authors:** Shoshana Rapley, Maldwyn J. Evans, Heather M. McGinness, Iain J. Gordon, Robert Heinsohn, Adrian D. Manning

**Affiliations:** ^1^ Fenner School of Environment and Society, the Australian National University Canberra Australian Capital Territory Australia; ^2^ Commonwealth Scientific and Industrial Research Organisation Canberra Australian Capital Territory Australia; ^3^ College of Science & Engineering, James Cook University Townsville Queensland Australia; ^4^ Research Division Central Queensland University Rockhampton Queensland Australia

**Keywords:** *Burhinus grallarius*, reinforcement, reintroduction, social network analysis, translocation

## Abstract

Animals released as part of reintroduction programmes are expected to benefit from social interactions with conspecifics from earlier releases, but empirical evidence is lacking. Using high‐resolution GPS tracking across a population of bush stone‐curlews ( *Burhinus grallarius* ), we investigated whether newly released animals (‘reinforcers’) interacted with previously released conspecifics (‘residents’), and whether these interactions influenced post‐release establishment. Post‐release outcomes for reinforcers varied with social group membership. Half the reinforcers integrated into the residents' social group and the other half did not. Reinforcers in the residents' social group had greater release‐site fidelity and faster roosting area establishment. Reciprocally, residents were attracted to the reinforcers, which enhanced resident roost‐site fidelity. We recommend developing translocation tactics that leverage social relationships to improve post‐release outcomes. These findings underscore the need to assess reinforcement outcomes at the population level, accounting for social dynamics between release cohorts.

## Graphical abstract

We investigated social interactions among release cohorts of reintroduced bush stone‐curlews. Newly released birds that had group membership with previously released residents had better outcomes: greater release site fidelity and faster roost establishment.

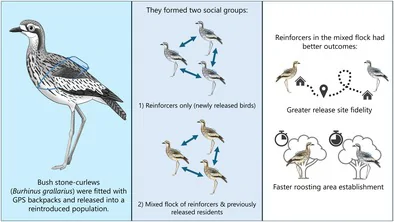

## 1 Introduction

Reintroductions—the translocation of species to areas in their historical range from which they have been extirpated—are increasingly used to restore lost biodiversity and ecological processes (Armstrong and Seddon 2008; Evans et al. 2023). Reintroduction projects typically release animals in stages (IUCN/SSC 2013), and reinforcing individuals may behave differently from founders released in the absence of conspecifics (Richardson and Ewen 2016). Given the altered social landscape, recognising this shift from reintroduction to reinforcement has important implications for translocation tactics and strategies (Batson et al. 2015). Furthermore, whether reinforcing individuals integrate or are excluded from the population depends on interactions with established individuals. Conspecific interactions mediate resource access, habitat use and mating opportunities (Reed and Dobson 1993; Armansin et al. 2020), directly influencing whether reinforcing individuals contribute to population‐level genetic and demographic outcomes, or are marginalised (Goldenberg et al. 2019).

While conspecific interactions *within* release cohorts are relatively well studied (Snijders et al. 2017), interactions *between* release cohorts remain poorly understood. Often consecutive release years are used as a proxy for conspecific effects, rather than measuring interactions directly (e.g., Dolev et al. 2002; Garnier et al. 2021; Sullivan et al. 2022). One exception is Cornelsen et al. (2025), who used social network analysis to examine associations between two greater bilby ( *Macrotis lagotis* ) release cohorts and found non‐preferential mixing, suggesting successful integration of reinforcers into the resident population. Beyond this, our understanding of how release cohorts interact draws largely from within‐cohort studies and reinforcements of wild populations. In these contexts, conspecific presence can facilitate establishment in several ways: residents may anchor new arrivals to release sites, transmit anti‐predator responses or migration strategies, reduce stress and provide habitat cues that shorten exploration time for suitable resources (Griffin et al. 2000; Swaisgood 2010; Scillitani et al. 2013; Jesmer et al. 2018; Garnier et al. 2021; Sullivan et al. 2022; Wilson et al. 2024). Conversely, residents can hinder reinforcement through territorial exclusion (Weilenmann et al. 2010; Ebrahimi and Bull 2014) and habitat copying can draw reinforcers into ecological traps when residents occupy poor‐quality habitat (Mihoub et al. 2009). These dynamics matter because they influence the fundamental goal of reinforcement: enhancing the viability of the population.

Beyond the limited understanding of between‐cohort interactions, how reinforcement success is measured also remains inconsistent. Success should be defined at the population level, given that reinforcement aims to enhance population size and genetic diversity. However, published studies often focus solely on the fate of the reinforcing individuals, neglecting population‐level outcomes (but see Champagnon et al. 2012). Previous studies have compared reinforcing individuals against residents as a metric for translocation success (Moehrenschlager and Macdonald 2003; Pinter‐Wollman et al. 2009; Bauder et al. 2014); however, the reciprocal effect—impact of reinforcers on residents—is rarely assessed or reported (e.g., data collected but not presented in Pille et al. 2018). In this study, we investigated the role of conspecific interactions in reinforcement outcomes. We reinforced a translocated population of bush stone‐curlews ( *Burhinus grallarius* ), a social, ground‐dwelling, nocturnal bird. We asked three questions: (1) To what extent are reinforcers integrated into the existing population? (2) Do reinforcers that socialise with residents show improved survival and establishment outcomes? (3) Do residents change their movement patterns in response to reinforcement? We hypothesised that reinforcers that formed social associations with residents would have higher survival rates and move more quickly through the phases of post‐release behavioural modification (*sensu* Berger‐Tal and Saltz 2014). Under this framework, individuals are predicted to shift from exploratory to exploitative movement patterns as they become familiar with their new environment. Finally, we hypothesised that residents would increase their core range size and distance travelled to compensate for increased competition for resources.

## 2 Methods

Translocation and monitoring procedures were approved by The Australian National University Animal Experimentation Ethics Committee (protocol A2022/14). Bush stone‐curlews were held at Mt. Rothwell under licence from the Victorian government (permit 13,306,138). Banding was approved by the Australian Bird and Bat Banding Scheme (banding project licence 1303/10).

### 2.1 Study Site

**FIGURE 1:**
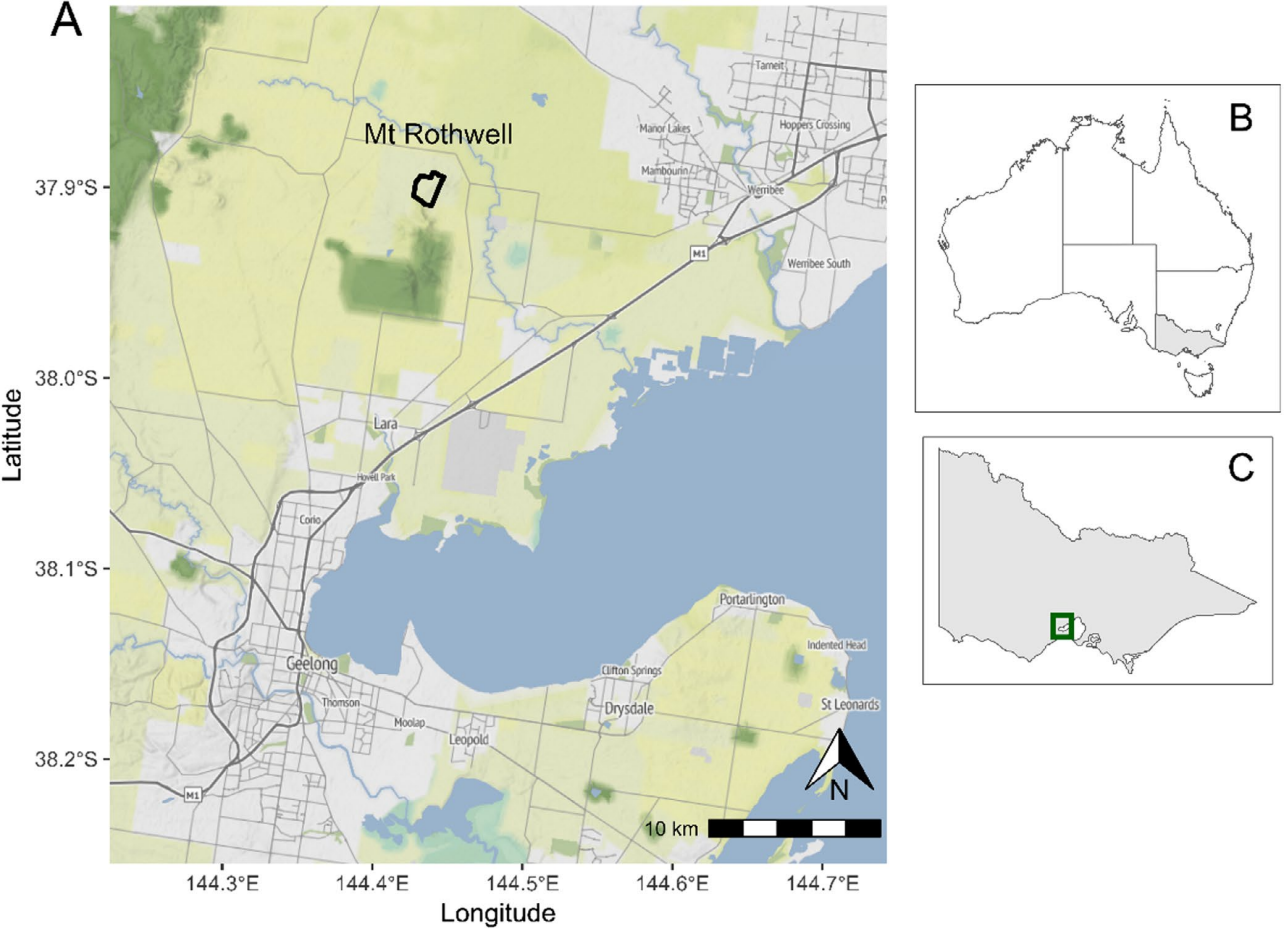
Study site location. (A) Map showing Mt. Rothwell captive breeding facility and fenced sanctuary in local area context. (B) Inset map of Australia with the state of Victoria shaded grey. (C) Inset map of the state of Victoria with the local area context shown as a green box. Terrain map from Stadia Maps.

Mt Rothwell Safe Haven (Figure 1; −37.895790, 144.438361; hereafter: Mt. Rothwell) is a captive breeding facility and 473 ha predator‐exclusion sanctuary in Victoria, on the traditional lands of the Wathawurrung people. Mt. Rothwell is owned and managed by the Odonata Foundation as part of a sanctuary network in southeast Australia. The sanctuary is fenced to exclude introduced mammalian predators, specifically the red fox ( *Vulpes vulpes* ) and feral cat ( *Felis catus* ).

Fenced sanctuaries exist on a continuum between fully wild and *ex situ* conservation populations, with their position determined by management intensity, size and permeability (Canessa et al. 2016; Neaves et al. 2025). The Mt. Rothwell sanctuary population occupies an intermediate‐wild position on this continuum: supplementary food is not provided, breeding is unmanaged, and the fence is permeable to some taxa (volant and climbing species). The sanctuary population functions as a quasi‐wild extension of the captive breeding facility. This intermediate position parallels the ‘*quasi in situ*’ concept in plant conservation, where living collections are managed under natural conditions to preserve genetic diversity and produce propagules for reintroduction (Volis 2017). Reintroduced populations are increasingly used as source populations for other translocation projects, often managed as a managed meta‐population (Pierson et al. 2023; Wilson et al. 2023).

The captive breeding population of bush stone‐curlews at Mt. Rothwell was established in 2019. Founding individuals were sourced from multiple captive populations across south‐east Australia. The facility contained 40 aviaries that were 4 × 5 m and 2 m high and had shelters, natural vegetation and woody debris to allow birds to display natural behaviours in accordance with species standards. Breeding pairs were housed together with their young until they reached subadult age, at which point birds were grouped with similarly‐aged individuals in groups of one to three per aviary. The birds were fed daily an appropriate protein‐based diet supplemented with insectivore rearing mix for dietary completeness. Additionally, the birds were fed live crickets once a week to simulate wild foraging behaviours. Water was provided *ad libitum* in bowls and baths.

### 2.2 Study Species

The bush stone‐curlew is a distinctive, medium‐sized (600–900 g), ground‐dwelling bird with predominantly nocturnal behaviour. During the day they roost under tree cover, particularly with leaf litter and woody debris and adopt a motionless, log‐like pose for camouflage, which is aided by their grey‐brown cryptic plumage. At night, they are active and vocal, running on long legs to hunt ground‐dwelling prey. These traits contribute both to the species' ecological uniqueness and to the challenges of monitoring populations. The species is monogamous with long‐term pair bonds, and breeding pairs typically hold small roosting territories (0.3–5 ha) year‐round, though some maintain larger ranges (10–70 ha) that contract seasonally (HANZAB 1993; Johnson and Baker‐Gabb 1994; Gates 2001). Outside the breeding season, unpaired and mobile individuals form loose flocks (Gates 2001). These flocks likely represent an important demographic phase bridging natal dispersal and pair formation (Price et al. 2018). The species is non‐migratory. The species formerly occurred across the Australian continent but has declined in the southern half of their range due to habitat loss and predation by introduced predators (DEC 2006; BirdLife International 2025). They are listed as Endangered in New South Wales (Biodiversity Conservation Act 2016) and Critically Endangered in Victoria (Flora and Fauna Guarantee Act 2004).

### 2.3 Translocation

We translocated 35 adult captive‐bred bush stone‐curlews from the Mt. Rothwell captive colony to Mt. Rothwell Zone 1–2 (210 ha subsection of the fenced sanctuary) in two stages: 16 birds were released in a pilot translocation in staged releases between October 2022 and January 2023, and a further 19 birds, along with one re‐released individual, were released in June 2023. We refer to these two cohorts as ‘residents’ (the first, primary cohort) and ‘reinforcers’ (the second, reinforcing cohort) following Wilson et al. (2024). One individual in the October 2022 release was returned to captivity 1 day post‐release due to erratic movements perceived as high‐risk; she was re‐released with the reinforcing cohort.

We based the translocation tactics on those developed at Mulligans Flat Woodland Sanctuary (Rapley 2020; Rapley et al. 2025). The full suite of translocation tactics (*sensu* Batson et al. 2015) is provided in Table S1; here we describe those relevant to this study. All translocated individuals were unpaired adults; the majority were 1–3 years old at the time of release, with three individuals aged 4–5 years. We selected a release location that was approximately central in the reserve to maximise distance from the fence (approx. 500 m) in an area with suitable vegetation cover for both roosting and foraging habitat. We wing clipped all translocated birds to reduce dispersal beyond the fence during the establishment phase when the risk of predation was high (Rapley et al. 2025). The primary release cohort was released gradually using a ‘drip‐feed’ tactic rather than as a single flock, to manage the risk and uncertainty of a first release at a new site. Releasing in small groups reduces conspicuousness to predators, exposes fewer individuals to risk early in the translocation and allows easier intervention if conditions change and released animals need to be recaptured (Rapley et al. 2025). Although this cohort was not released simultaneously, all individuals were established by the time of the reinforcing release. The reinforcing cohort was also drip‐fed released, but over days rather than weeks.

Health checks were conducted prior to release and at one, three and six months post‐release where possible; not all individuals could be recaptured at every interval due to the challenges of capturing free‐living birds. Birds were captured by hand, with a gas‐powered net launcher, or in a mist net. Checks included body weight and condition scoring, and a full physical examination for signs of injury or illness. Birds exhibiting suspected health issues were referred for veterinary assessment. Prior to release, all birds were treated for intestinal parasites and feather mites.

Ten of the reinforcers were translocated to a secondary site in August 2023 for a separate conservation translocation, which marked the end of the study period. Reinforcers were moved to the secondary site to test a ‘stepping‐stone’ release tactic, in which individuals are staged through a population with conspecifics before being moved to a new site, rather than translocated directly from captivity (Lloyd et al. 2019).

### 2.4 GPS Telemetry

We used Ornitrack‐20 solar‐powered GPS‐GSM tracking devices manufactured by Ornitela. Devices weighed 20 g, representing a mean of 2.7% body mass (range: 2.1%–3.4%). We fitted GPS units with a backpack‐style harness made of teflon ribbon with a weak‐link at the keel (per the design used in Rapley 2020; McGinness et al. 2024). We previously used this design on bush stone‐curlews at Mulligans Flat Woodland Sanctuary without adverse effects (Rapley 2020). Devices recorded GPS fixes at 60‐s intervals, reducing to 180‐s intervals below 75% battery and 300‐s intervals below 50% battery. Data were transmitted via GSM over the 3G cellular network every 24 h.

### 2.5 Data Analyses

We archived GPS tracking data on Movebank and analysed data in R version 4.4.1 (R Core Team 2024). We produced plots using the package ggplot2 (Wickham et al. 2016) and maps using the package ggmap (Kahle and Wickham 2013).

#### 2.5.1 GPS Data Pre‐Processing

We pre‐processed the GPS tracking data following recommendations by Gupte et al. (2022). We filtered the data to the study period and the fenced area. We retained fixes with a satellite count ≥ 4 and HDOP ≤ 2. We filtered out biologically unrealistic speed and turning angles (based on the 99th percentile of movement data; defined here as a maximum speed of 1.9 m/s and a maximum turning speed of 0.5 m/s for angles > 90°). We then applied a rolling average location filter to remove outliers (fixes > 100 m from the median position of a moving window of seven fixes) and applied median smoothing with a moving window of three fixes with the function atl_median_smooth() from the atlastools package (Gupte et al. 2022). We thinned the data to the coarsest common sampling interval among the birds, using the function track_resample() from the amt package (Signer et al. 2019) with a rate of 10 min and a tolerance of 2 min.

#### 2.5.2 Social Network Analyses

We conducted all network analyses using the package tidygraph (Pedersen 2025). We used social network analysis to identify group co‐membership following reinforcement. For each dyad, we calculated a proximity score and used this score as the weight in a weighted network framework. To calculate the dyadic proximity scores, we used the function prox() from the package wildlifeDI (Long et al. 2022) with a time criterion of 330 s (half the mean sampling rate) and a distance criterion of 30 m (based on average GPS error and visual contact distance). We selected proximity analysis because it incorporates interaction frequency (Farine and Whitehead 2015) and has low type I error rates (Long et al. 2014). While this approach infers interactions from spatiotemporal proximity rather than observing them directly, our dataset was highly complete, with fine temporal resolution and complete coverage of the population. This trade‐off between detailed information on individual interactions and broad population coverage is common in GPS‐based social network analysis (Farine and Whitehead 2015; He et al. 2023). We detected community structure using the weighted spinglass method, which performs well for small dense networks such as ours (Yang et al. 2016).

#### 2.5.3 Post‐Release Behavioural Modification Metrics

We calculated the following post‐release behavioural modification metrics per day: distance travelled, distance between consecutive roosts, distance from the release site, and core range size. We calculated distance travelled using the function steps() from the package amt (Signer et al. 2019). We identified daily roost locations by calculating the centroid of diurnal GPS fixes for each day using the function kmeans() from base R. We calculated the distance between consecutive daily roost sites using the function st_distance() from the package sf (Pebesma 2018). We calculated the distance between the daily roost site and the release site using the function st_distance(). We calculated core range using the function kernelUD() from the package adehabitatHR (Calenge 2006) and defined core range as the 50% kernel utilisation distribution (KUD). We used the core range instead of the broader 90% KUD home range because the 90% KUD was constrained by the fence area, resulting in boundary artefacts. For reinforcers, we calculated post‐reinforcement movement metrics from the date of the last reinforcer release until 3 August 2023 (55 days), when capture of reinforcers for translocation to a secondary site began. For residents, the window began from the date of the first reinforcer release (58 days), as residents could be influenced by conspecifics from the first new arrival. We applied the same 58‐day window pre‐reinforcement for comparison.

#### 2.5.4 Statistical Analyses

We performed generalised linear mixed model (GLMM) fitting with the package glmmTMB (Brooks et al. 2017). Hypothesis one (social integration) was addressed by the social network analysis described above. To address hypothesis two, we used social group membership as a predictor variable for reinforcement outcomes. We modelled each movement metric using a GLMM with social group membership (mixed, exclusive, or resident) as a fixed effect, with linear and quadratic time terms and their interactions with social group to test for differences between the groups over the post‐reinforcement period. Residents were included as the reference level to allow comparison of reinforcer social groups against the established population, in addition to with each other. Individual was included as a random effect to account for repeated measures.

To address hypothesis three, we tested whether resident movements differed pre‐ and post‐reinforcement. We modelled each movement metric using a GLMM with a binary period variable (pre/post reinforcement), linear and quadratic time terms and their interactions with period, allowing us to test both immediate shifts at reinforcement and changes in trajectory between periods. Individual was included as a random effect to account for repeated measures.

To correct skewness (Zuur et al. 2010), we applied a square root transformation to distance moved from the release site, and a log transformation to distance moved between roosts and core range size. Additionally, we tested the effect of group membership and time (pre‐ or post‐release) on reinforcer body mass. Following data transformation, residuals approximated Gaussian distributions, so we specified Gaussian error structures for all models. We used the function dredge() from the package MuMIn (Bartoń 2015) to compare all subsets of each model for the movement metrics. We selected the most parsimonious model (fewest terms) within ΔAICc ≤ 2 of the top‐ranked model (Burnham and Anderson 2002). Full model selection tables are reported in Tables S2 and S3, and fixed effect estimates of the selected models are reported in Tables S4 and S5. We assessed model fit using residual diagnostics, implemented with the package performance (Lüdecke et al. 2021). We used the function emtrends() from the package emmeans (Lenth 2025) to perform pairwise comparisons of marginal linear trends among social groups, with Tukey adjustment for multiple comparisons. Estimated marginal trends are reported in Table S6. Where response variables were transformed for analysis, we report model estimates on the transformed scale. We present back‐transformed predictions in the figures for ease of interpretation.

We used survival analysis to test whether survival differed (a) between cohorts and (b) between reinforcers of different social groups. We tested survival as a time‐to‐event response using a Cox proportional hazards model in the package survival (Therneau 2024). We assessed survival to 55 days post‐release for both cohorts to enable direct comparison across the minimum common tracking period.

## 3 Results

### 3.1 Survival

Of the 35 birds released, 27 were alive at the end of the study. The rate of survival to 55 days post‐release was 87% for the residents (*n* = 16; one predation event by a bird of prey and one death attributed to acute gastroenteritis) and 95% for the reinforcers (*n* = 19; one death attributed to acute gastroenteritis). The difference in survival between cohorts was not significant (likelihood ratio test: χ^2^ = 0.37, df = 1, *p* = 0.542), though statistical power was limited by few mortality events. Five additional residents died after the initial 55‐day period, all from fox predation outside the fenced area. At the time of the reinforcing release, eight residents remained.

### 3.2 Social Organisation

**FIGURE 2:**
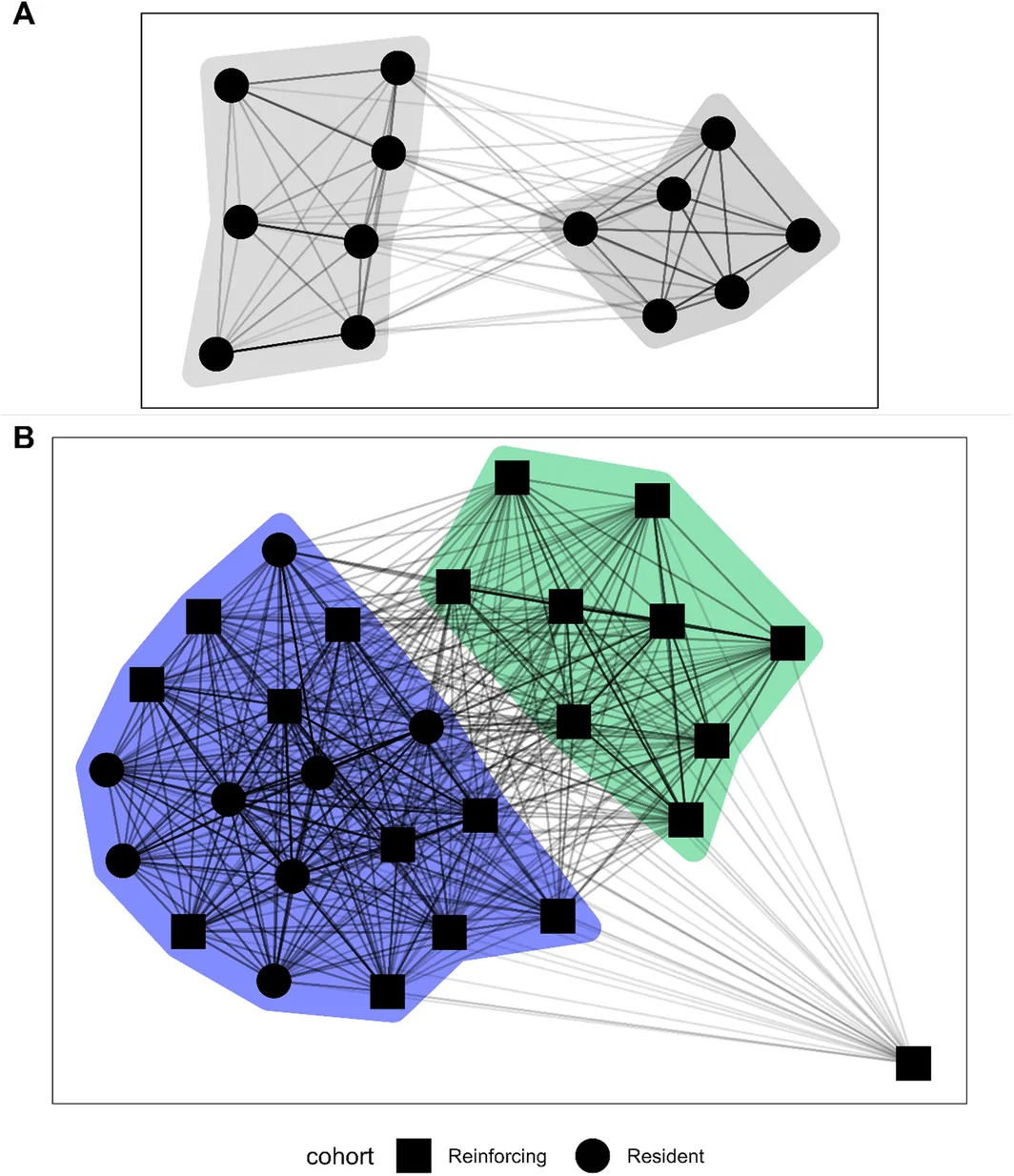
Social network of bush stone‐curlews ( *Burhinus grallarius* ) before (A) and after (B) a reinforcing translocation. Shaded polygons indicate social group community structure (colours correspond to group assignments in Figure 3). Edges (connections) are weighted by proximity scores between dyads, where edge opacity and linewidth increase with proximity scores. Resident (from the first release cohort) nodes are shown as circles while reinforcer (from the second release cohort) nodes are shown as squares.

Prior to reinforcement, the residents had a continuous social network (edge density: 87.2%) comprised of two groups (Figure 2A). Following reinforcement, the population formed a continuous social network (edge density: 100%) comprising two groups and one lone individual (Figure 2B). Of the two groups, one consisted exclusively of reinforcers (*n* = 9, henceforth ‘exclusive reinforcers’) and the other was a mixed group of reinforcers (*n* = 10, henceforth ‘mixed reinforcers’) and residents (*n* = 8).

### 3.3 Reinforcer Outcomes by Social Group Membership

We found no difference in survival between mixed reinforcers and exclusive reinforcers (likelihood ratio test: χ^2^ = 0.58, df = 1, *p* = 0.448). Only one reinforcer died during the study period: a member of the mixed social group whose death was attributed to acute gastroenteritis.

**FIGURE 3:**
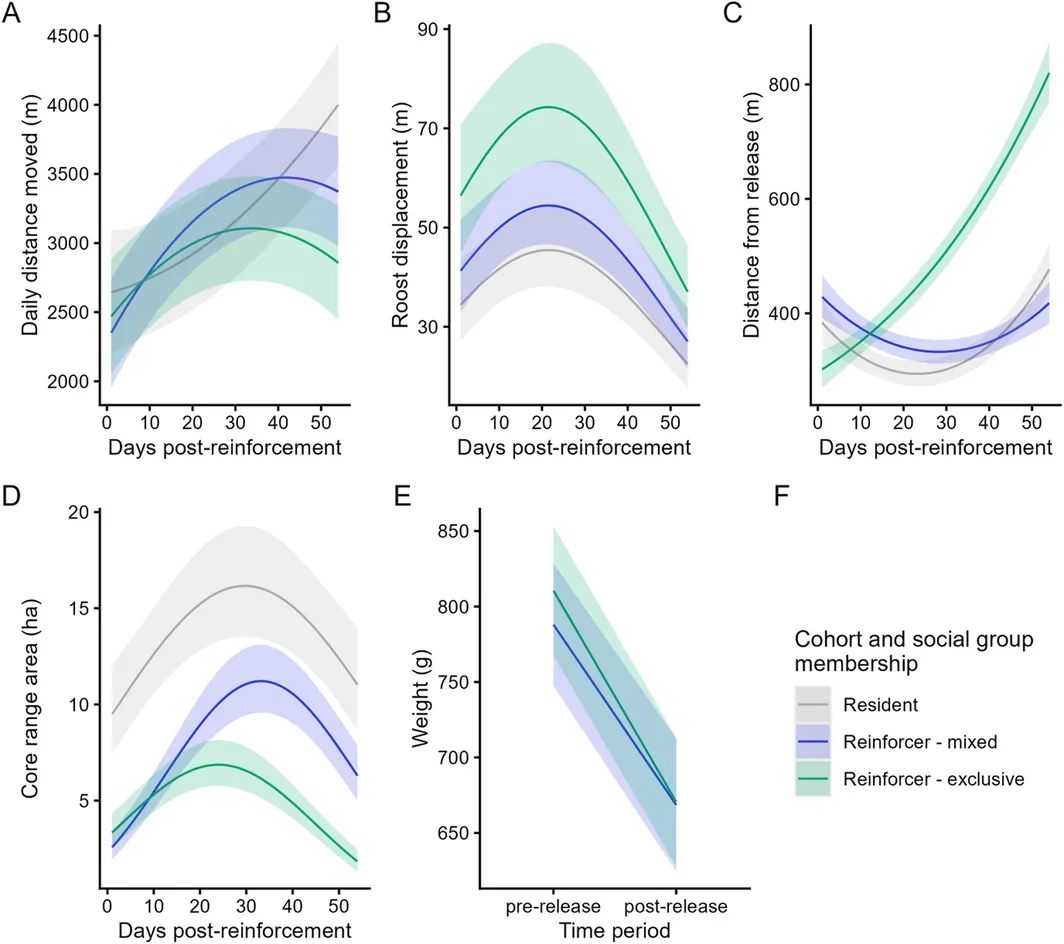
Post‐release outcomes for translocated bush stone‐curlews ( *Burhinus grallarius* ) by cohort and social group membership. Lines are predicted values and bands are 95% confidence intervals. Cohorts are residents (first, pilot release) and reinforcers (secondary, reinforcing release). Reinforcers self‐selected into two social group membership of exclusive (co‐membership exclusively with other reinforcers) or mixed (co‐membership with reinforcers and residents). (A) Daily distance moved in meters. (B) Distance between consecutive roosts in meters. (C) Distance from release‐site in meters. (D) Core range area (defined as 50% kernel utilisation distribution) in hectares. (E) Weight pre‐ and post‐release in grams, for reinforcers only. (F) Legend.

Daily distance moved by reinforcers increased over time (399.01 ± 40.58 SE, *p* < 0.001; Figure 3A). Both reinforcer groups showed significant negative quadratic terms compared to residents (mixed: −245.46 ± 59.41 SE, *p* < 0.001; exclusive: −226.78 ± 60.27 SE, *p* < 0.001), indicating that the increase in distance moved decelerated and declined for reinforcers while continuing to increase for residents. Pairwise comparison of marginal trends showed that exclusive reinforcers had a significantly lower rate of increase than both residents (*p* < 0.001) and mixed reinforcers (*p* < 0.001). The difference between residents and mixed reinforcers was not significant (*p* = 0.159).

Roost displacement initially increased before declining (linear: −0.12 ± 0.03, *p* < 0.001; quadratic: −0.16 ± 0.04, *p* < 0.001; Figure 3B). Exclusive reinforcers moved significantly further between consecutive roost sites than did residents (0.48 ± 0.11 SE, *p* < 0.001), whereas mixed reinforcers did not differ from residents (0.18 ± 0.11 SE, *p* = 0.104).

**FIGURE 4:**
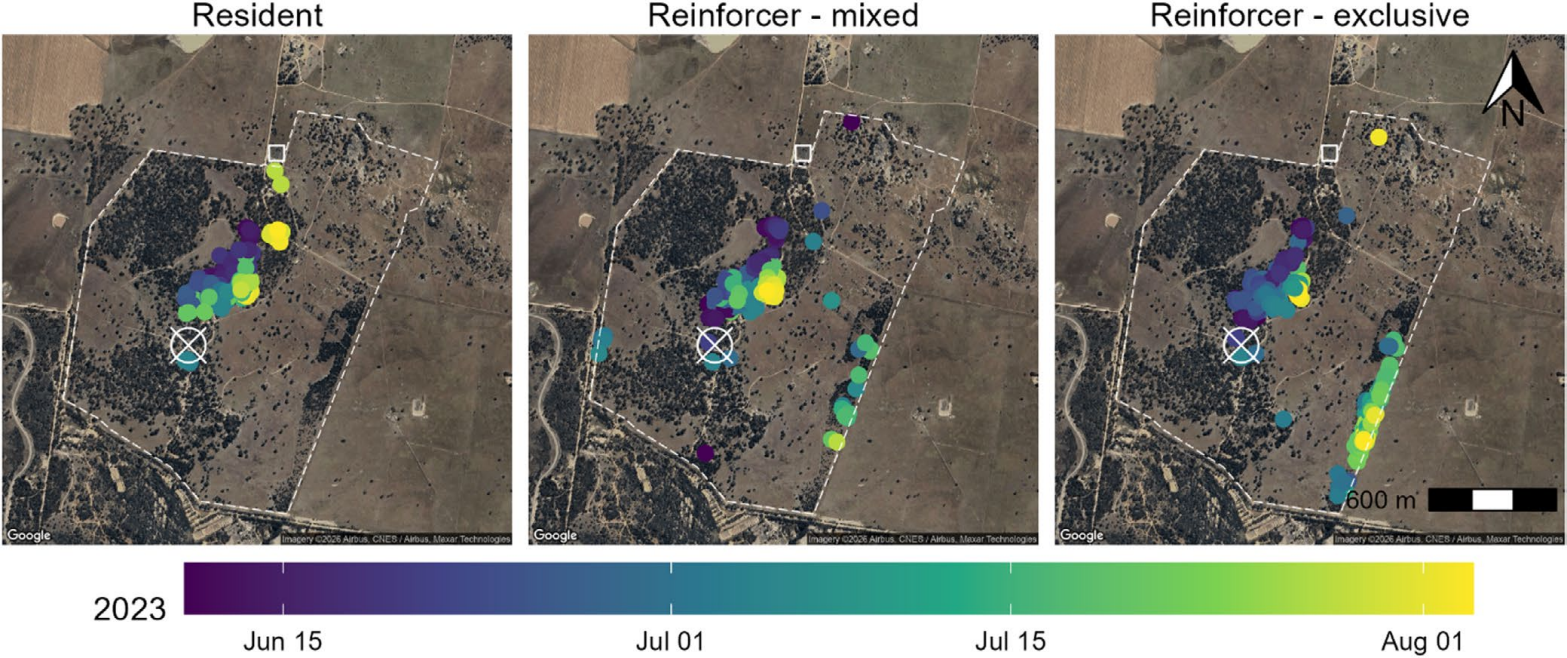
Daily roost locations of bush stone‐curlews ( *Burhinus grallarius* ) by cohort and social group membership. Cohorts are residents (first, pilot release) and reinforcers (secondary, reinforcing release). Reinforcers self‐selected into social group membership of exclusive (co‐membership exclusively with other reinforcers) or mixed (co‐membership with reinforcers and residents). Roosts are coloured by date, transitioning from purple to yellow with increased time elapsed following the release of the reinforcing cohort. Dashed line indicates the predator exclusion fence. The white crosshairs symbol indicates the release location. The white square indicates the captive breeding facility location. Background satellite imagery from Google Maps.

Distance from the release site increased over time (0.66 ± 0.16 SE, *p* < 0.001; Figure 3C). Pairwise comparisons of marginal trends showed that all three groups differed significantly from each other (all *p* ≤ 0.002). Relative to residents, exclusive reinforcers dispersed from the release site 3.6 times faster than did mixed reinforcers (trend differences: 0.170 vs. 0.047). Mixed reinforcers and residents settled in the central woodland, while exclusive reinforcers moved over time from the central woodland to the eastern boundary (Figure 4).

Core range size was largest for residents, smallest for exclusive reinforcers and intermediate for mixed reinforcers (Figure 3D). Both groups of reinforcers had significantly smaller core ranges than the residents (mixed: −0.38 ± 0.11 SE, *p* < 0.001; exclusive: −0.79 ± 0.12 SE, *p* < 0.001). Mixed reinforcers increased core range size relative to residents (0.17 ± 0.04 SE, *p* < 0.001), whereas exclusive reinforcers decreased core range size relative to residents (−0.17 ± 0.04 SE, *p* < 0.001).

Reinforcers in the mixed social group lost less weight (mean loss 109.25 g, 13.8% of starting weight) than did reinforcers in the exclusive social group (mean loss 140.0 g, 17.3% of starting weight), but this effect was not significant (−20.55 ± 28.22 SE, *p* = 0.467; Figure 3E).

### 3.4 Resident Response to Reinforcement

**FIGURE 5:**
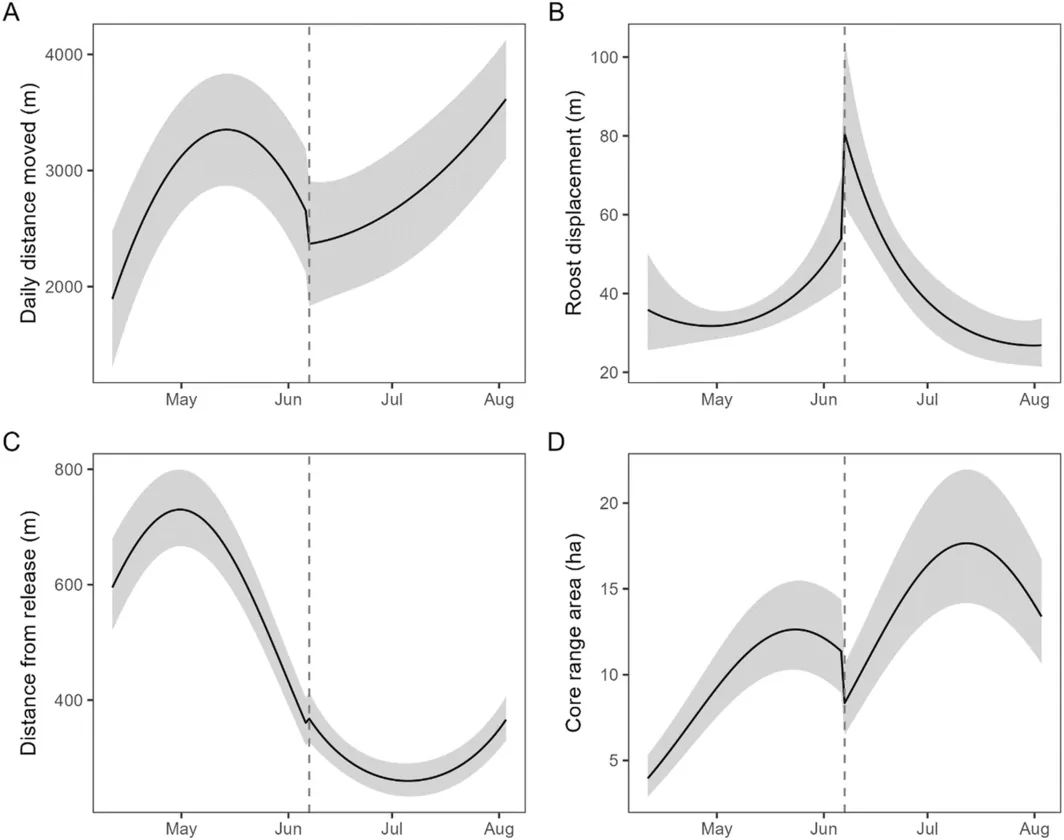
Effect of reinforcing translocation on resident reintroduced bush stone‐curlews ( *Burhinus grallarius* ). Lines are predicted values and bands are 95% confidence intervals. Dashed line indicates the date of reinforcement. (A) Daily distance moved in meters. (B) Distance between consecutive roosts in meters. (C) Distance from release‐site in meters. (D) Core range area (defined as 50% kernel utilisation distribution) in hectares.

Reinforcement caused an immediate reduction in distance moved by residents (−505.8 ± 199.9, *p* = 0.011), followed by an increase that exceeded pre‐reinforcement levels (linear interaction: 2254.4 ± 508.4, *p* < 0.001; quadratic interaction: 1818.4 ± 279.1, *p* < 0.001; Figure 5A). Reinforcement caused an immediate increase in roost displacement (0.649 ± 0.162, *p* < 0.001), followed by a reversal of the pre‐reinforcement trend (linear interaction: −2.229 ± 0.597, *p* < 0.001; Figure 5B), indicating an increase in roost‐site fidelity. Distance from the release site declined over time (−1.315 ± 0.090 SE, *p* < 0.001) with no immediate shift at reinforcement (−0.005 ± 0.059 SE, *p* = 0.933). Distance from the release site was already declining pre‐reinforcement, and this trend stabilised and slightly reversed post‐reinforcement (linear interaction: 0.514 ± 0.151, *p* < 0.001; quadratic interaction: 1.062 ± 0.083, p < 0.001; Figure 5C). Reinforcement caused an immediate reduction in core range size (−0.493 ± 0.084, *p* < 0.001), followed by an increase that exceeded pre‐reinforcement levels (linear interaction: 1.817 ± 0.307, p < 0.001; Figure 5D). Residents moved up to 3 km beyond the fenced sanctuary boundary.

## 4 Discussion

We examined the outcomes of reinforcement from three perspectives: the population as a whole, the reinforcing individuals and the resident individuals. Social network analysis has previously been used to assess the integration of translocated individuals into wild populations (Poirier and Festa‐Bianchet 2018), but has not previously been applied to assess interactions between sequentially released cohorts within a reintroduction program.

### 4.1 Population‐Level Effect of Reinforcement

The reinforcement achieved its intended effect, increasing the population from eight to 27 individuals. Although interactions occurred between all reinforcer and resident dyads, community membership was partitioned, with only 10 of 19 reinforcers integrating into the resident community. This partitioning potentially has consequences for population demography, although we did not monitor the population through to reproduction. Bush stone‐curlews may take multiple years to breed following translocation. Since social structures can constrain mating opportunities (Armansin et al. 2020) and bush stone‐curlews form lifelong monogamous pairs (HANZAB 1993), the partitioning of reinforcers into separate social groups could limit mate choice. Alternatively, exclusive reinforcers may integrate given additional time; for example, it took one year for translocated bighorn sheep ( *Ovis canadensis* ) to integrate into a wild population (Poirier and Festa‐Bianchet 2018), though notably, the bighorn sheep were integrating into an established wild population rather than a recently translocated one.

Acute gastrointestinal disease accounted for two of the three early deaths, one in each cohort. Translocation stress can compromise immune function in the immediate post‐release period, potentially explaining the early disease‐related mortality (Dickens et al. 2010). Whether the disease was present subclinically prior to release and manifested under translocation stress, or was acquired post‐release, remains unknown. A similar phenomenon was observed in kākāpō ( *Strigops habroptilus* ) where translocation stress increased susceptibility to a disease outbreak (Gartrell et al. 2005).

### 4.2 Reinforcer Movement Post Release Was Moderated by Social Group Membership

Social group membership shaped post‐release outcomes for reinforcing bush stone‐curlews. Reinforcers that held group co‐membership with residents did not exhibit a distinct exploration phase before settling on a roost area. Instead, they displayed a high degree of fidelity to the roost area in the central woodland, where the residents preferentially roosted. By contrast, the exclusive reinforcers increased their distance between consecutive roosts for approximately 30 days before stabilising and moved to the eastern boundary of the reserve, the maximum distance available from the release site. Conspecifics can act as a ‘magnet’ for newly translocated individuals (Garnier et al. 2021). This magnet effect fits within the broader theory of attraction and avoidance in habitat selection, which predicts that naïve newcomers select habitat based on conspecific cues (Stamps 1991; Muller et al. 1997). Our results suggest that the magnet effect is not universal; rather, its strength depends on the nature of social interactions between cohorts. Whether the exclusive reinforcers chose to move away from the release area or were territorially excluded remains unclear. Territorial exclusion can be density dependent (Deredec and Courchamp 2007) and the mixed group of 18 may have represented the maximum group size or density supported by local resource availability. Alternatively, exclusion and attraction could have been driven entirely by familiarity and social interactions. Familiarity reduced territoriality among members of a translocated cohort of toutouwai ( *Petroica australis* ) in New Zealand (Armstrong 1995). Regardless of the mechanism, exclusion from the resident community likely has negative fitness consequences for reinforcers. Indeed, the habitat selected by the exclusive reinforcers along the eastern fence was less suitable than the central woodland area, with less tree cover and denser shrubs compared with the species' preferred habitat (Rapley 2020). Future research could investigate whether pre‐release traits predict which individuals integrate with residents and thereby benefit from the magnet effect.

Although social groups differed in their spatial establishment patterns, both groups of reinforcers shared similar temporal patterns of post‐release movement behaviour regardless of their community membership. Reinforcers from both social groups exhibited a gradual increase in distance travelled and core range size, which peaked at approximately 30 days post‐release before declining. This pattern is consistent with previous studies in which movement initially increases, followed by a decline and stabilisation (Clapp et al. 2014; Sullivan et al. 2022). It also aligns with the theoretical prediction that translocated animals shift from exploration to exploitation as they become familiar with their new environment (Berger‐Tal and Saltz 2014). All reinforcers had significantly smaller core ranges than did residents, which contrasts with previous studies where translocated animals typically have larger core ranges than do residents (Sullivan et al. 2015; Wilson et al. 2024). Smaller core ranges for the reinforcers may reflect the wing‐clipping intervention, which temporarily restricts flight capability. Wing clipping reduces hyperdispersal into high‐predation risk areas beyond the fence during the establishment period and improves survival in bush stone‐curlews (Rapley 2020; Rapley et al. 2025). Wing clipping may slow post‐release behavioural modification by constraining movement; however, this trades off against reduced predation risk, as predation is one of the leading causes of translocation failure (Sheean et al. 2012; Berger‐Tal et al. 2020).

Reinforcers from both social groups lost weight post‐release. Some weight loss upon release into a more demanding environment is expected, particularly for captive‐bred animals whose starting weights were at the upper end of the species' range. Bush stone‐curlews weigh 570–810 g (Schodde and Mason 1980) and the mean starting weight for reinforcers was 799 g. Additionally, seasonal weight loss is expected due to the higher energetic demands of winter; birds in the captive breeding facility also lost weight over the same time period (unpublished data, this study). Members of the exclusive social group lost on average 21 g more than members of the mixed social group. While this effect was not statistically significant, it could be biologically relevant: an additional 2.5% mass loss during winter could influence health outcomes. Starvation is a known cause of reintroduction failure for the species (Kemp and Roshier 2016), so tactics that influence weight maintenance may meaningfully impact survival in other reintroduction contexts.

A limitation of our approach was that the birds self‐selected their groups. The individual traits that led mixed reinforcers to associate with residents may also have predisposed them to better post‐release outcomes, independent of the social interaction itself. A comparable pattern was observed in translocated hihi ( *Notiomystis cincta* ) where individuals that formed new social bonds after translocation were more likely to survive, but whether sociability itself was the mechanism was unclear (Franks et al. 2020). We also do not know whether self‐selection of groups was due to pre‐release social bonds. However, translocated juvenile hihi did not maintain their pre‐release bonds (Franks et al. 2020). Future research should investigate whether pre‐release individual traits such as boldness, sociability, or prior social bonds predict post‐release social group membership. Most research on conspecific attraction in birds has focused on migratory passerines (Valente et al. 2021), and whether similar mechanisms operate in resident, ground‐dwelling species such as bush stone‐curlews remains unknown.

### 4.3 Effect of Reinforcement on Residents

Prior to reinforcement, the residents had two social groups, which collapsed into one larger group also including reinforcers. This restructuring challenges existing theory about translocation impacts on social structures. Translocation has been theorised to disrupt social groups because the act of translocation removes external influences that held social groups together (Franks et al. 2020). However, in our study we saw disruption to social groups in the recipient site, where there is no change to the external influences, just the social dynamic. Consequently, we suggest that translocation itself was the disruptive force, rather than a change in context.

Residents increased their core range size and daily distance moved, aligning with our hypothesis that they would expand their space use to compensate for increased competition for resources. Both metrics had plateaued prior to reinforcement, dropped briefly during the reinforcement event, and then increased beyond pre‐reinforcement levels. Core range size expanded for approximately one month before stabilising at a new equilibrium, indicating the reinforcement caused a temporary disruption. Daily distance moved, by contrast, continued to increase throughout the post‐reinforcement period. These increases may reflect the need to accommodate a larger group, as the addition of 10 mixed reinforcers more than doubled the resident group size. This aligns with the ecological‐constraints model, which predicts that larger groups need to travel farther to obtain the resources needed to match their energetic demands (Ganas and Robbins 2005; Teichroeb and Sicotte 2009; Papageorgiou and Farine 2020). The effect of reinforcement on residents' spatial behaviour likely varies with the social organisation of the species. In territorial species, home range size typically decreases with increasing population density (Schradin et al. 2010; Balluffi‐Fry et al. 2025), whereas in social species, home range size typically increases with group size (Ganas and Robbins 2005). However, some social species have a non‐linear relationship between home range and group size, due to the challenges of maintaining social cohesion in larger groups (Papageorgiou and Farine 2020). Bush stone‐curlews exhibit both territorial defence by breeding pairs and large social groups comprising non‐breeding ‘floater’ individuals, and these groups can form and collapse seasonally (HANZAB 1993; Gates 2001; Price et al. 2018). Consequently, predicting the effect of reinforcement may be particularly challenging in species like bush stone‐curlews that exhibit variable social structures, as outcomes may depend on the social landscape of the recipient population at the time of release.

Residents stabilised their distance from the release site and increased roost‐site fidelity following reinforcement. Just as residents acted as ‘magnets’ for newly translocated individuals (Garnier et al. 2021), reinforcers may have reciprocally anchored residents to the release area. This attraction could help address a critical challenge in bush stone‐curlew reintroductions. Five of the eight residents were predated by foxes after dispersing beyond the fence, all after moulting their trimmed primaries and regaining flight capability. This pattern of post‐moult dispersal‐driven attrition has also been observed in previous bush stone‐curlew translocations (Rapley 2020). A reinforcing release, therefore, could be used to mitigate the dispersal‐driven attrition that usually occurs around the moult window. Most dispersal management tactics target newly released individuals during the immediate post‐release period (Batson et al. 2015; Bilby and Moseby 2024). Wing clipping effectively reduced dispersal at this stage but appears to have delayed rather than eliminated dispersal risk, with attrition concentrated instead around the moult window. Nevertheless, 90‐day post‐release survival for the resident cohort in this study was 70%, compared with only 27% in the first bush stone‐curlew translocation at Mulligans Flat Woodland Sanctuary where wing clipping was not used (Rapley et al. 2025), further demonstrating the importance of this tactic in reducing early post‐release mortality. While post‐moult attrition remains a concern, strategically timed reinforcement releases offer one potential tactic to mitigate dispersal at this stage. In future, tactics targeting the moult window warrant investigation.

## 5 Conclusion

Social group membership shaped reinforcement outcomes, with consequences for both reinforcing and resident individuals. Reinforcers that integrated into the resident social community showed more conservative range expansion and greater release‐site fidelity. Future research should investigate whether pre‐release individual traits predict social integration, which could inform the selection of individuals for reinforcement releases. We also provide evidence for a reciprocal magnet effect, whereby reinforcers anchored residents to the release area. Strategically timed reinforcing releases could therefore serve as an anchoring tactic to mitigate dispersal‐driven attrition in residents. Finally, our population‐level approach revealed effects on residents that individual‐level assessment would have missed, underscoring the need to evaluate reinforcement outcomes across the entire population.

## Author Contributions

**Shoshana Rapley:** conceptualization (equal), data curation (lead), formal analysis (lead), funding acquisition (equal), investigation (lead), methodology (lead), software (equal), validation (equal), visualization (lead), writing – original draft (lead), writing – review and editing (lead). **Maldwyn J. Evans:** conceptualization (equal), formal analysis (supporting), methodology (equal), supervision (equal), writing – review and editing (equal). **Heather M. McGinness:** conceptualization (equal), methodology (equal), supervision (equal), writing – review and editing (equal). **Iain J. Gordon:** conceptualization (equal), methodology (equal), supervision (equal), writing – review and editing (equal). **Robert Heinsohn:** conceptualization (equal), methodology (equal), supervision (equal), writing – review and editing (equal). **Adrian D. Manning:** conceptualization (equal), funding acquisition (equal), methodology (equal), project administration (equal), resources (lead), supervision (equal), writing – review and editing (equal).

## Funding

This study was supported by the Fenner School of Environment and Society at the Australian National University and the Odonata Foundation. SR was supported by the Australian Government Research Training Program (AGRTP) PhD Scholarship.

## Conflicts of Interest

The authors declare no conflicts of interest.

## Supporting information


**Table S1:** Translocation tactics (Sensu Batson et al. 2015) to promote post‐release performance in translocated bush stone‐curlews ( *Burhinus grallarius* ) at Mt. Rothwell. The tactics are organised by focus (animal or environment) and groups of related tactics. The tactic implementation describes how the tactic was implemented in this study. The tactic aim describes the intended purpose of implementing the selected tactic.
**Table S2:** Model selection results for linear mixed models of post‐release movement behaviours among social groups of translocated bush stone‐curlews (Burhinus grallarius). + indicates the term was included in the candidate model. The selected model (fewest terms within ΔAICc ≤ 2 of the top‐ranked model) is shown in bold. All models included individual as a random intercept and social group as a fixed effect. Social group is not shown as it was retained in all candidate models. AICc: = Akaike information criterion corrected for small samples.
**Table S3:** Model selection results for linear mixed models of resident bush stone‐curlew (Burhinus grallarius) movement behaviours before and after reinforcement. + indicates the term was included in the candidate model. The selected model (fewest terms within ΔAICc ≤ 2 of the top‐ranked model) is shown in bold. All models included individual as a random intercept. AICc: = Akaike information criterion corrected for small samples.
**Table S4:** Fixed effect estimates from linear mixed models comparing post‐release movement behaviours among social groups of translocated bush stone‐curlews (Burhinus grallarius). Social group contrasts are relative to residents. Time was scaled (centred and standardised). All models included individual as a random intercept.
**Table S5:** Fixed effect estimates from linear mixed models of resident bush stone‐curlew (Burhinus grallarius) movement behaviours before and after reinforcement. Period contrasts are relative to pre‐reinforcement. Time was scaled (centred and standardised). All models included individual as a random intercept.
**Table S6:** Estimated marginal trends from pairwise Tukey‐adjusted comparisons of elapsed time trends (slopes) between social reinforcement groups. Contrasts for distance from release site and core range are on the transformed scale (square‐root and log, respectively).

## Data Availability

Data and code used in this study are available at https://github.com/coexistence‐conservation‐lab/mtr‐bsc‐reinforce.
