# Supplementary material for: Social Group Membership With Residents Mediates Reinforcement Outcomes for a Translocated Bird
Source: Ecol Evol. 2026 Aug 20;16(8):e74136. doi: 10.1002/ece3.74136 (PMC13491385; doi:10.1002/ece3.74136)
Supplement: Supplementary file 1 [file ECE3-16-e74136-s001.docx]

# Supplementary materials

**Supplementary Table S1:** Translocation tactics (Sensu Batson *et al.* 2015) to promote post-release performance in translocated bush stone-curlews (*Burhinus grallarius*) at Mt Rothwell. The tactics are organised by focus (animal or environment) and groups of related tactics. The tactic implementation describes how the tactic was implemented in this study. The tactic aim describes the intended purpose of implementing the selected tactic.

| **Tactic focus**  **& group** | **Tactic type** | **Tactic implementation** | **Tactic aim/justification** |
| --- | --- | --- | --- |
| Animal selection | Physiological | Good/excellent condition. | Sufficient body condition pre-release to buffer against initial post-release weight loss. |
|  | Demographic | Even sex ratio. | Monogamous species- allow for maximum reproductive potential. |
|  |  | Adults in first few years of life. | Individuals that no longer require parental care but minimise time spent in captivity (and potential habituation). |
| Animal pre-conditioning | Behavioural | Live food (crickets) provisioned weekly. | Encourage wild feeding behaviours. |
|  | Social | Housed in same or adjacent aviaries. | Aid conspecific identification and encourage group formation. |
|  | Experiential | Provisioning of natural substrate with leaf litter and woody debris. | Enable practice camouflaging (a key anti-predator behaviour for the species). |
|  | Health | Treatment for feather mites | Maximise quality of feathers and reduce parasite transfer into natural environment. |
|  | Physiological | Wing-clipping. | Prevent hyper-dispersal (beyond fence) in crucial period post-release where naivety is highest. |
| Post-release animal management | Intervention | Post-release monitoring for health and behaviour. Return to captivity as needed. | Minimise mortality. |
| Environmental release design | Temporal configuration | Drip-feed release approach, released in consecutive smaller groups rather than large flock. | Manage risk and uncertainty during first translocation to a new site. |
|  | Release timing | Released on dusk. | Species is nocturnal and forages at night. Minimise risk of predation. |
|  | Immediate release | Released from transport crates after a short (15 minute) settling period. | Reduce stress. Soft release found unnecessary (and potentially maladaptive learning) in previous releases. |
|  | Passive release | Leave transport crates of own accord (instead of e.g., being taken out and released). | Minimise stress and maximise physiological calm for decision making. |
| Environmental selection | Suitability | Presence of habitat features identified in Rapley (2020). | Suitable habitat key predictor of translocation success. |
|  | Threat control | Pre-existing predator-exclusion fencing. | Minimise risk of predation by introduced predators. |

**Supplementary Table S2**: Model selection results for linear mixed models of post-release movement behaviours among social groups of translocated bush stone-curlews (*Burhinus grallarius*). + indicates the term was included in the candidate model. The selected model (fewest terms within ΔAICc ≤ 2 of the top-ranked model) is shown in bold. All models included individual as a random intercept and social group as a fixed effect. Social group is not shown as it was retained in all candidate models. AICc: = Akaike information criterion corrected for small samples.

| **Time** | **Time^2^** | **Social × Time** | **Social × Time^2^** | **df** | **logLik** | **AICc** | **ΔAICc** | **Weight** |
| --- | --- | --- | --- | --- | --- | --- | --- | --- |
| **Daily distance moved** | | | | | | | | |
| **+** | **+** | **+** | **+** | **11** | **-11390.3** | **22802.8** | **0.00** | **1.000** |
| + | + | + | — | 9 | -11400.3 | 22818.6 | 15.88 | < 0.001 |
| + | + | — | + | 9 | -11404.9 | 22827.8 | 25.09 | < 0.001 |
| + | — | + | — | 8 | -11407.4 | 22830.8 | 28.09 | < 0.001 |
| + | + | — | — | 7 | -11415.6 | 22845.3 | 42.57 | < 0.001 |
| + | — | — | — | 6 | -11422.8 | 22857.6 | 54.85 | < 0.001 |
| — | + | — | + | 8 | -11474.4 | 22965.0 | 162.20 | < 0.001 |
| — | + | — | — | 6 | -11484.4 | 22980.8 | 178.03 | < 0.001 |
| — | — | — | — | 5 | -11489.1 | 22988.3 | 185.55 | < 0.001 |
| **Roost displacement** | | | | | | | | |
| **+** | **+** | **—** | **+** | 9 | -2200.8 | 4419.7 | 0.00 | 0.480 |
| + | + | — | — | **7** | **-2203.4** | **4420.8** | **1.12** | **0.274** |
| + | + | + | + | 11 | -2199.9 | 4421.9 | 2.23 | 0.157 |
| + | + | + | — | 9 | -2202.5 | 4423.1 | 3.40 | 0.088 |
| — | + | — | + | 8 | -2208.1 | 4432.2 | 12.56 | 0.001 |
| — | + | — | — | 6 | -2210.8 | 4433.7 | 13.98 | < 0.001 |
| + | — | — | — | 6 | -2213.6 | 4439.3 | 19.58 | < 0.001 |
| + | — | + | — | 8 | -2212.7 | 4441.6 | 21.91 | < 0.001 |
| — | — | — | — | 5 | -2221.7 | 4453.4 | 33.71 | < 0.001 |
| **Distance from release site** | | | | | | | | |
| **+** | **+** | **+** | **+** | **11** | **-3648.3** | **7318.8** | **0.00** | **0.984** |
| + | + | + | — | 9 | -3654.5 | 7327.1 | 8.30 | 0.016 |
| + | — | + | — | 8 | -3684.5 | 7385.0 | 66.24 | < 0.001 |
| + | + | — | + | 9 | -3779.8 | 7577.7 | 258.95 | < 0.001 |
| + | + | — | — | 7 | -3783.1 | 7580.2 | 261.45 | < 0.001 |
| + | — | — | — | 6 | -3810.1 | 7632.2 | 313.42 | < 0.001 |
| — | + | — | + | 8 | -3867.0 | 7750.2 | 431.42 | < 0.001 |
| — | + | — | — | 6 | -3869.4 | 7750.9 | 432.17 | < 0.001 |
| — | — | — | — | 5 | -3897.0 | 7804.0 | 485.23 | < 0.001 |
| **Core range (50% KUD)** | | | | | | | | |
| **+** | **+** | **+** | **+** | **11** | **-1313.3** | **2648.8** | **0.00** | **0.977** |
| + | + | + | — | 9 | -1319.1 | 2656.3 | 7.49 | 0.023 |
| + | + | — | + | 9 | -1350.2 | 2718.5 | 69.68 | < 0.001 |
| — | + | — | + | 8 | -1353.7 | 2723.5 | 74.70 | < 0.001 |
| + | + | — | — | 7 | -1355.9 | 2725.8 | 77.04 | < 0.001 |
| — | + | — | — | 6 | -1359.4 | 2730.8 | 82.00 | < 0.001 |
| + | — | + | — | 8 | -1404.3 | 2824.6 | 175.84 | < 0.001 |
| + | — | — | — | 6 | -1438.6 | 2889.2 | 240.40 | < 0.001 |
| — | — | — | — | 5 | -1440.5 | 2891.0 | 242.20 | < 0.001 |

**Supplementary Table S3:** Model selection results for linear mixed models of resident bush stone-curlew (*Burhinus grallarius*) movement behaviours before and after reinforcement. + indicates the term was included in the candidate model. The selected model (fewest terms within ΔAICc ≤ 2 of the top-ranked model) is shown in bold. All models included individual as a random intercept. AICc: = Akaike information criterion corrected for small samples.

| **Time** | **Time^2^** | **Post × Time** | **Post × Time^2^** | **df** | **logLik** | **AICc** | **ΔAICc** | **Weight** |
| --- | --- | --- | --- | --- | --- | --- | --- | --- |
| **Daily distance moved** | | | | | | | | |
| **+** | **+** | **+** | **+** | **8** | **-7915.0** | **15846.2** | **0.00** | **1.000** |
| + | + | — | + | 7 | -7924.8 | 15863.6 | 17.43 | < 0.001 |
| + | + | + | — | 7 | -7935.8 | 15885.7 | 39.50 | < 0.001 |
| — | + | — | + | 6 | -7938.7 | 15889.5 | 43.27 | < 0.001 |
| + | — | + | — | 6 | -7945.3 | 15902.8 | 56.59 | < 0.001 |
| — | + | — | — | 6 | -7951.1 | 15914.2 | 68.00 | < 0.001 |
| + | — | — | — | 5 | -7958.6 | 15927.3 | 81.13 | < 0.001 |
| — | — | — | — | 5 | -7973.3 | 15956.6 | 110.43 | < 0.001 |
| — | — | — | — | 4 | -7982.0 | 15972.0 | 125.86 | < 0.001 |
| **Roost displacement** | | | | | | | | |
| + | + | + | + | 8 | -1484.6 | 2985.4 | 0.00 | 0.523 |
| **+** | **+** | **+** | **—** | **7** | **-1485.9** | **2985.9** | **0.49** | **0.410** |
| + | — | + | — | 6 | -1488.8 | 2989.7 | 4.27 | 0.062 |
| — | + | — | — | 5 | -1493.1 | 2996.2 | 10.79 | 0.002 |
| + | + | — | — | 6 | -1492.8 | 2997.7 | 12.32 | 0.001 |
| — | + | — | + | 6 | -1493.0 | 2998.0 | 12.60 | 0.001 |
| + | + | — | + | 7 | -1492.4 | 2999.0 | 13.57 | 0.001 |
| — | — | — | — | 4 | -1504.7 | 3017.5 | 32.09 | < 0.001 |
| + | — | — | — | 5 | -1504.3 | 3018.8 | 33.33 | < 0.001 |
| **Distance from release site** | | | | | | | | |
| **+** | **+** | **+** | **+** | **8** | **-172.2** | **360.6** | **0.00** | **0.991** |
| + | + | — | + | 7 | -178.0 | 370.1 | 9.47 | 0.009 |
| + | + | + | — | 7 | -248.0 | 510.1 | 149.46 | < 0.001 |
| + | — | + | — | 6 | -250.1 | 512.3 | 151.69 | < 0.001 |
| + | + | — | — | 6 | -262.1 | 536.3 | 175.71 | < 0.001 |
| — | + | — | + | 6 | -286.3 | 584.7 | 224.10 | < 0.001 |
| — | + | — | — | 5 | -299.6 | 609.2 | 248.61 | < 0.001 |
| + | — | — | — | 5 | -331.6 | 673.3 | 312.67 | < 0.001 |
| — | — | — | — | 4 | -359.5 | 726.9 | 366.33 | < 0.001 |
| **Core range (50% KUD)** | | | | | | | | |
| **+** | **+** | **+** | **+** | 8 | -861.7 | 1739.5 | 0.00 | 0.683 |
| + | + | + | — | **7** | **-863.5** | **1741.1** | **1.53** | **0.317** |
| — | + | — | + | 6 | -877.0 | 1766.2 | 26.66 | < 0.001 |
| + | + | — | + | 7 | -876.6 | 1767.3 | 27.74 | < 0.001 |
| + | + | — | — | 6 | -880.7 | 1773.5 | 33.97 | < 0.001 |
| + | — | + | — | 6 | -888.8 | 1789.7 | 50.18 | < 0.001 |
| + | — | — | — | 5 | -897.2 | 1804.5 | 64.95 | < 0.001 |
| — | + | — | — | 5 | -904.5 | 1819.1 | 79.58 | < 0.001 |
| — | — | — | — | 4 | -918.3 | 1844.7 | 105.13 | < 0.001 |

**Supplementary Table S4**: Fixed effect estimates from linear mixed models comparing post-release movement behaviours among social groups of translocated bush stone-curlews (*Burhinus grallarius*). Social group contrasts are relative to residents. Time was scaled (centred and standardised). All models included individual as a random intercept.

| **Fixed effect** | **Estimate** | **SE** | **z** | **p** |
| --- | --- | --- | --- | --- |
| **Daily distance moved**  Gaussian, untransformed; n = 1402, 27 individuals | | | | |
| Intercept | 3081.8 | 207.2 | 14.88 | < 0.001 |
| Time | 399.0 | 40.58 | 9.83 | < 0.001 |
| Time^2^ | 80.05 | 44.92 | 1.78 | 0.075 |
| Social [mixed] | 245.8 | 276.8 | 0.89 | 0.374 |
| Social [exclusive] | -0.848 | 283.3 | -0.00 | 0.998 |
| Social [mixed] × Time | -98.71 | 53.80 | -1.84 | 0.067 |
| Social [exclusive] × Time | -284.9 | 54.24 | -5.25 | < 0.001 |
| Social [mixed] × Time^2^ | -245.5 | 59.41 | -4.13 | < 0.001 |
| Social [exclusive] × Time^2^ | -226.8 | 60.27 | -3.76 | < 0.001 |
| **Roost displacement**  Gaussian, log-transformed; n = 1400, 27 individuals | | | | |
| Intercept | 3.818 | 0.089 | 42.71 | < 0.001 |
| Time | -0.121 | 0.031 | -3.87 | < 0.001 |
| Time^2^ | -0.158 | 0.035 | -4.54 | <0.001 |
| Social [mixed] | 0.177 | 0.109 | 1.63 | 0.104 |
| Social [exclusive] | 0.483 | 0.111 | 4.36 | < 0.001 |
| **Distance from release site**  Gaussian, square-root transformed; n = 1402, 27 individuals | | | | |
| Intercept | 17.23 | 0.337 | 51.17 | < 0.001 |
| Time | 0.658 | 0.163 | 4.03 | < 0.001 |
| Time^2^ | 1.208 | 0.182 | 6.63 | < 0.001 |
| Social [mixed] | 1.013 | 0.444 | 2.28 | 0.022 |
| Social [exclusive] | 4.707 | 0.453 | 10.38 | < 0.001 |
| Social [mixed] × Time | -0.734 | 0.218 | -3.37 | < 0.001 |
| Social [exclusive] × Time | 2.646 | 0.220 | 12.01 | < 0.001 |
| Social [mixed] × Time^2^ | -0.396 | 0.242 | -1.63 | 0.102 |
| Social [exclusive] × Time^2^ | -0.862 | 0.246 | -3.50 | < 0.001 |
| **Core range (50% KUD)**  Gaussian, log-transformed; n = 1404, 27 individuals | | | | |
| Intercept | 2.839 | 0.085 | 33.46 | < 0.001 |
| Time | 0.040 | 0.031 | 1.31 | 0.192 |
| Time^2^ | -0.147 | 0.034 | -4.28 | < 0.001 |
| Social [mixed] | -0.381 | 0.112 | -3.38 | < 0.001 |
| Social [exclusive] | -0.787 | 0.115 | -6.84 | < 0.001 |
| Social [mixed] × Time | 0.170 | 0.041 | 4.16 | < 0.001 |
| Social [exclusive] × Time | -0.165 | 0.041 | -4.00 | < 0.001 |
| Social [mixed] × Time^2^ | -0.144 | 0.045 | -3.17 | 0.002 |
| Social [exclusive] × Time^2^ | -0.129 | 0.046 | -2.81 | 0.005 |
| **Weight**  Gaussian, untransformed; n = 36, 19 individuals | | | | |
| Intercept | 788.0 | 20.67 | 38.13 | < 0.001 |
| Social [exclusive] | 22.56 | 30.03 | 0.75 | 0.453 |
| Status [post-release] | -119.5 | 20.42 | -5.85 | < 0.001 |
| Social [exclusive] × Status | -20.55 | 28.22 | -0.73 | 0.467 |

**Supplementary Table S5**: Fixed effect estimates from linear mixed models of resident bush stone-curlew (*Burhinus grallarius*) movement behaviours before and after reinforcement. Period contrasts are relative to pre-reinforcement. Time was scaled (centred and standardised). All models included individual as a random intercept.

| **Fixed effect** | **Estimate** | **SE** | **z** | **p** |
| --- | --- | --- | --- | --- |
| **Daily distance moved**  Gaussian, untransformed; n = 953, 10 individuals | | | | |
| Intercept | 2855.0 | 261.8 | 10.91 | < 0.001 |
| Time | -2104.5 | 302.5 | -6.96 | < 0.001 |
| Time^2^ | -1480.1 | 191.3 | -7.74 | < 0.001 |
| Post | -505.8 | 199.9 | -2.53 | 0.011 |
| Post × Time | 2254.4 | 508.4 | 4.43 | < 0.001 |
| Post × Time^2^ | 1818.4 | 279.1 | 6.52 | < 0.001 |
| **Roost displacement**  Gaussian, log-transformed; n = 955, 10 individuals | | | | |
| Intercept | 3.916 | 0.108 | 36.42 | < 0.001 |
| Time | 0.926 | 0.272 | 3.40 | < 0.001 |
| Time^2^ | 0.401 | 0.166 | 2.41 | 0.016 |
| Post | 0.649 | 0.162 | 4.01 | < 0.001 |
| Post × Time | -2.229 | 0.597 | -3.74 | < 0.001 |
| **Distance from release site**  Gaussian, log-transformed; n = 955, 10 individuals | | | | |
| Intercept | 6.016 | 0.053 | 112.59 | < 0.001 |
| Time | -1.315 | 0.090 | -14.64 | < 0.001 |
| Time^2^ | -0.591 | 0.057 | -10.40 | < 0.001 |
| Post | -0.005 | 0.059 | -0.08 | 0.933 |
| Post × Time | 0.514 | 0.151 | 3.40 | < 0.001 |
| Post × Time^2^ | 1.062 | 0.083 | 12.81 | < 0.001 |
| **Core range (50% KUD)**  Gaussian, log-transformed; n = 955, 10 individuals | | | | |
| Intercept | 2.565 | 0.105 | 24.35 | < 0.001 |
| Time | -0.523 | 0.140 | -3.74 | < 0.001 |
| Time^2^ | -0.616 | 0.085 | -7.22 | < 0.001 |
| Post | -0.493 | 0.084 | -5.89 | < 0.001 |
| Post × Time | 1.817 | 0.307 | 5.93 | < 0.001 |

**Supplementary Table S6:** Estimated marginal trends from pairwise Tukey-adjusted comparisons of elapsed time trends (slopes) between social reinforcement groups. Contrasts for distance from release site and core range are on the transformed scale (square-root and log, respectively).

| **Contrast** | **Estimate** | **SE** | **t** | **df** | **p** |
| --- | --- | --- | --- | --- | --- |
| **Daily distance moved** | | | | | |
| Resident – Mixed | 6.33 | 3.45 | 1.84 | 1391 | 0.159 |
| Resident – Exclusive | 18.27 | 3.48 | 5.25 | 1391 | < 0.001 |
| Mixed – Exclusive | 11.94 | 3.23 | 3.69 | 1391 | < 0.001 |
| **Distance from release site** | | | | | |
| Resident – Mixed | 0.047 | 0.014 | 3.37 | 1389 | 0.002 |
| Resident – Exclusive | −0.170 | 0.014 | −12.01 | 1389 | < 0.001 |
| Mixed – Exclusive | −0.217 | 0.013 | −16.34 | 1389 | < 0.001 |
| **Core range (50% KUD)** | | | | | |
| Resident – Mixed | −0.011 | 0.003 | −4.16 | 1393 | < 0.001 |
| Resident – Exclusive | 0.011 | 0.003 | 4.00 | 1393 | < 0.001 |
| Mixed – Exclusive | 0.022 | 0.002 | 8.70 | 1393 | < 0.001 |
